# Pre-profiling factors influencing serum microRNA levels

**DOI:** 10.1186/1472-6890-14-27

**Published:** 2014-06-21

**Authors:** Sara A MacLellan, Calum MacAulay, Stephen Lam, Cathie Garnis

**Affiliations:** 1 Department of Integrative Oncology, British Columbia Cancer Research Centre, Vancouver, BC, Canada; 2 Department of Surgery, University of British Columbia, Vancouver, BC, Canada

**Keywords:** MicroRNA, Cancer, Biomarker, Serum, miR-99a-5p, miR-139-5p

## Abstract

**Background:**

MicroRNAs (miRNAs) are non-coding RNAs that negatively regulate gene expression by preventing the translation of specific mRNA transcripts. Recent studies have shown that miRNAs are stably expressed in human serum samples, making them good candidates for the non-invasive detection of disease. However, before circulating miRNAs can be used reliably as biomarkers of disease, the pre-measurement variables that may affect serum miRNA levels must be assessed.

**Methods:**

In this study we used quantitative RT-PCR to examine the effect of hemolysis, fasting, and smoking on the levels of 742 miRNAs in the serum of healthy individuals. We also compared serum miRNA profiles of samples taken from healthy individuals over different time periods to assess normal serum miRNA fluctuations.

**Results:**

We have found that mechanical hemolysis of blood samples can significantly alter serum miRNA quantification and have identified 162 miRNAs that are significantly up-regulated in hemolysed serum samples. Conversely, fasting and smoking were demonstrated to not have a significant effect on the overall serum miRNA profiles of healthy individuals. The serum miRNA profiles of matched samples taken from individuals over varying time periods showed a high correlation and no miRNAs were significantly differentially expressed in these samples further suggesting the utility of serum miRNAs as biomarkers of disease. Taking the above results into consideration, we have identified miR-99a-5p and miR-139-5p as novel endogenous controls for serum miRNA studies due to their consistency across all sample sets.

**Conclusion:**

These results identify important pre-profiling factors that should be taken into consideration when identifying endogenous controls and candidate biomarkers for circulating miRNA studies.

## Background

MicroRNAs (miRNAs) are small, endogenous, non-coding RNAs that negatively regulate gene expression by interfering with the translation of target mRNAs [1]. Currently, over 1600 mature miRNA sequences have been identified in humans (miRBase, Release 19.0, http://www.mirbase.org). Each miRNA can target multiple mRNAs and it has been estimated that over 30% of the human genome is regulated by miRNAs [2]. Consequently, miRNAs have been shown to be involved in many cancer-associated cellular processes including development, cell proliferation, apoptosis, fat metabolism, and cell differentiation [3-8].

Until recently, miRNAs were thought to be unstable in the extracellular environment but two independent studies published in 2008 confirmed that miRNAs were present in a stable form in clinical samples of serum and plasma [9,10]. These groups also demonstrated that circulating miRNAs exhibit altered expression under certain disease states and could potentially be used as diagnostic and/or prognostic biomarkers. Since 2008, a number of studies have identified candidate miRNA biomarkers for numerous conditions including drug-induced liver injury [11], acute myocardial infarction [12], and various cancers [13-16]. However, studies identifying miRNA biomarkers for the same disease have shown variable results. Due to the lack of a full understanding of circulating miRNAs, the factors that may affect their quantification are difficult to predict. Some of this variability may be attributed to pre-profiling sources such as patient lifestyle and sample collection methods. Other possible sources of variability in circulating miRNA profiles are analytical differences such as the quantification platform or normalization methods used [17].

Before circulating miRNAs can reliably be used as biomarkers of disease, the pre-profiling factors that may contribute to inconsistent miRNA quantification must be identified and the variability of circulating miRNAs in healthy individuals must be determined. Recent studies have shown that the majority of miRNAs found in serum and plasma are also present in blood cells and that hemolysis during sample collection or processing may affect the miRNA profile of the sample [10,18,19]. In particular, circulating miRs 451a, 16-5p, 24-3p, 15b-5p, 223-3p, 486-5p, 150-5p and 92a-3p have all been shown to be inconsistently expressed in healthy individuals due to their presence in blood cells [20,21]. Additionally, studies examining a large number of miRNAs in serum and plasma have shown that many of the miRNAs most commonly expressed in these samples show an altered expression in samples exhibiting blood cell contamination [22,23]. Therefore, the effects of mechanical hemolysis on whole micronome serum quantification require further examination in patient-matched samples to ensure that potential biomarkers are not compromised by blood cell contamination.

Furthermore, it has been suggested that lipid intake before blood draw may interfere with miRNA extraction leading to a variable profile [24] and that miRNAs present in food may influence serum miRNA profiles of healthy individuals [25]. However, there have been no studies examining the effects of fasting status on miRNA expression profiles.

Lifestyle may also affect serum miRNA expression. A number of recent studies have focused on the use of circulating miRNAs as lung cancer biomarkers [10,16,26]. Because ~85% of lung cancers in men and 45% in women are caused by smoking [27], there is a need to examine the effects of smoking on circulating miRNA profiles before these molecules can be used as lung cancer biomarkers. One study has shown that serum levels of miR-625* are significantly lower in non-small cell lung cancer patients who smoke compared to those who do not smoke [28]. However, there have been no studies comparing the serum miRNA levels of healthy smokers to healthy non-smokers. Other unknown factors or lifestyle changes may influence the serum miRNA profiles of healthy individuals leading to a change in these profiles over time.

In this study, we examined the effect of hemolysis, fasting, and smoking on serum miRNA profiles of healthy individuals and compared serum miRNA profiles of samples taken from healthy individuals over different time periods. Our results contribute to a better understanding of the pre-analytical factors that may influence serum miRNA profiles.

## Methods

### Ethics statement

Written consent was obtained from all patients prior to obtaining blood samples. Use of human specimens in this work was approved by the University of British Columbia ethics board (H10-02846).

### Sample collection

To determine the effects of hemolysis on serum miRNA profiles, two vials of blood were drawn from 10 healthy volunteers. One vial was lysed immediately after collection by passing the blood through a 20 gauge needle several times. This method has been shown to mimic mechanical hemolysis due to improper blood collection or specimen preparation [29]. To assess serum miRNA variability in healthy individuals and to determine the effects of fasting on serum miRNA profiles, blood samples were drawn from 7 healthy volunteers one hour after eating a fatty meal and again, three weeks later, after fasting overnight. Serum triglyceride levels were measured for each sample using a Triglyceride Assay Kit (Cayman Chemical Company, Ann Arbor, MI, USA). To determine the effects of smoking on serum miRNA profiles, blood samples were collected from 10 current smokers and from 10 age and sex-matched non-smokers without evidence of disease. Finally, to examine changes in serum miRNA levels over time two samples were collected from 12 healthy volunteers over various time periods. All blood samples were drawn into serum separator vacutainer tubes and kept at room temperature for 30 minutes to allow clotting. Clotted samples were centrifuged at room temperature (15 minutes, 1500 rcf), aliquoted, and frozen at -80°C within 2 hours of collection.

### Hemoglobin concentration

Serum absorbance levels were measured spectrophotometrically using a NanoDrop ND1000 spectrophotometer (NanoDrop Technologies). The hemoglobin concentration of each sample was calculated using the Harboe method [30] with the following formula:

$$\begin{array}{l} \mathrm{Hemoglobin}\;\left(g/l\right)\;=\;\left(167.2\;A_{415}-\;83.6\;A_{380}-\;83.6\;A_{450}\right)\\ \;\times \;1/1000 \end{array}$$

where A_415_, A_380_ and A_450_ are the absorption at 415, 380 and 450 nm respectively.

### RNA purification

Total RNA was extracted from 200 μL of serum using miRNeasy Mini Kit (Qiagen) as previously described [31]. For some samples a synthetic spike-in consisting of 20 fmol of synthetic *C. elegans* miR-39-3p (cel-miR-39-3p; Qiagen, Toronto, ON, Canada) miRNA was added to each sample after a 5 minute incubation in QIAzol Lysis Reagent.

### miRNA quantification by qRT-PCR

The levels of 742 miRNAs (target sequences available at http://www.exiqon.com/plate-layout-files V2.0) were quantified using miRNome real-time PCR panels (Version V2M, Exiqon Inc., Woburn, MA) as previously described [31]. Due to low RNA yields in serum, the concentration of purified RNA could not be reliably measured, hence fixed volumes of eluted RNA (19.2 μL for 768 reactions) were used for miRNA expression assays as per the manufacturers recommendation. For samples profiled with a synthetic spike-in, 1μl of LNA™ control cel-mir-39-3p primer (Exiqon) was added to two empty wells on each panel.

### Data analysis

Ct values and ROX reference dye normalization were calculated using Viia7 Software (Applied Biosystems). All assays were inspected for distinct melting curves – those with >1 T_m_ and those detected within 5 Ct’s of the negative control (Ct >35) were excluded from analysis. qRT-PCR results were exported to GenEx (MultiD Analyses AB) and normalized using inter-plate calibrators on the miRNA Ready-to-Use panels. There are currently no standard endogenous controls for serum miRNA studies [32]. Therefore we selected an endogenous control suitable for each sample set as described in results. Because less abundant miRNAs showed high variability, miRNAs detected in < 80% of case or control samples were excluded from analyses. To compare matched samples, a fold-change analysis was used (2^(case∆Ct - control∆Ct)^, ∆Ct = Raw endogenous control Ct – Raw assay Ct) and to identify the most significantly deregulated miRNAs a 3-fold change cut-off was applied (selection of fold-change (FC) cut-off explained in results). Average-linkage Pearson correlation hierarchical clustering was calculated and displayed using Genesis software (http://www.genome.tugraz.at). The Mann-Whitney U test was conducted using Statistica Software (Statsoft™, Tulsa, OK). All *P*-values were corrected for multiple testing using the Benjamini and Hochberg method.

## Results

### Determining the best fold-change (FC) cut-off value

**Figure 1:**
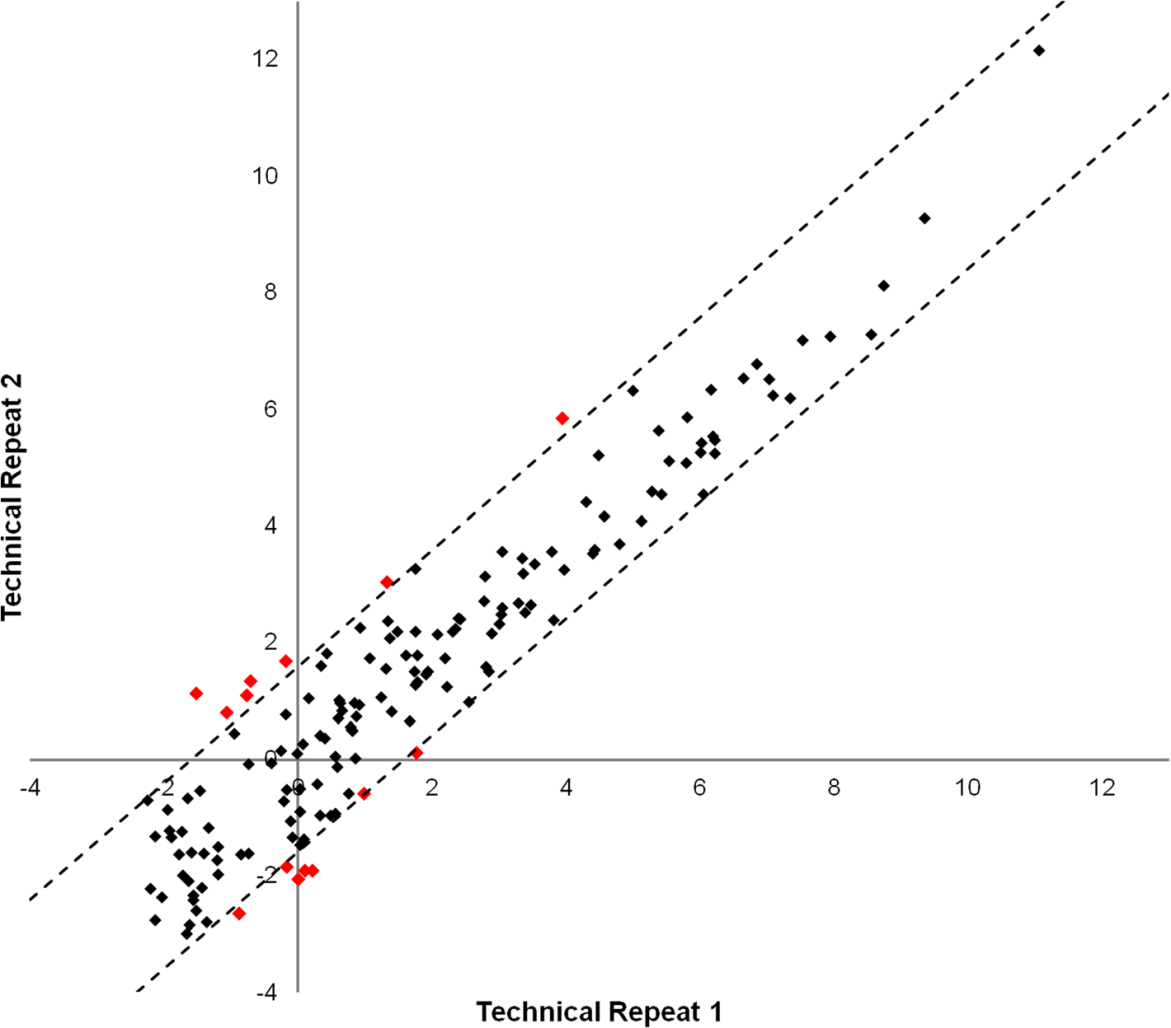
**Technical Repeat.** A scatter plot of the normalized Ct values for 157 miRNAs from a technical repeat experiment in which serum collected from the same patient was aliquoted and subjected to two separate RNA purification and miRNA quantification steps. The dotted line represents the 3-fold difference boundary between the two samples. The 14 miRNAs showing a ≥ 3-fold difference are highlighted in red.

To determine the appropriate FC cut-off to use when identifying significantly differentially expressed miRNAs via a fold-change analysis, we examined the consistency of our assay by analyzing a technical repeat of our quantification methods. Serum collected from a healthy non-smoker was aliquoted into two separate vials (200 μl each) and two separate RNA purification and miRNA quantification experiments were conducted. The results were normalized to miR-99a-5p and 139-5p and a fold-change analysis was conducted. Of the 157 miRNAs detected in both samples, 48 (31%) miRNAs showed ≥2-fold difference between the two samples, 14 (9%) showed a ≥3-fold difference, and 5 (3%) of miRNAs showed a ≥4-fold difference. Because serum samples contain a relatively low concentration of RNA we anticipated that much of the observed variation was resulting from miRNAs with high Ct values. Indeed, 38 of the 48 miRNAs that exhibited a ≥2-fold difference had raw Ct values >30 and all 48 miRNAs had a raw Ct value >28. Because the improvement between a 3-fold and 4-fold FC cut-off was modest in comparison to the improvement between a 2-fold and 3-fold FC cut-off, we decided to consider miRNAs with a ≥3-fold difference as significant in further analyses (Figure 1).

### The effects of hemolysis on serum miRNA levels

**Figure 2:**
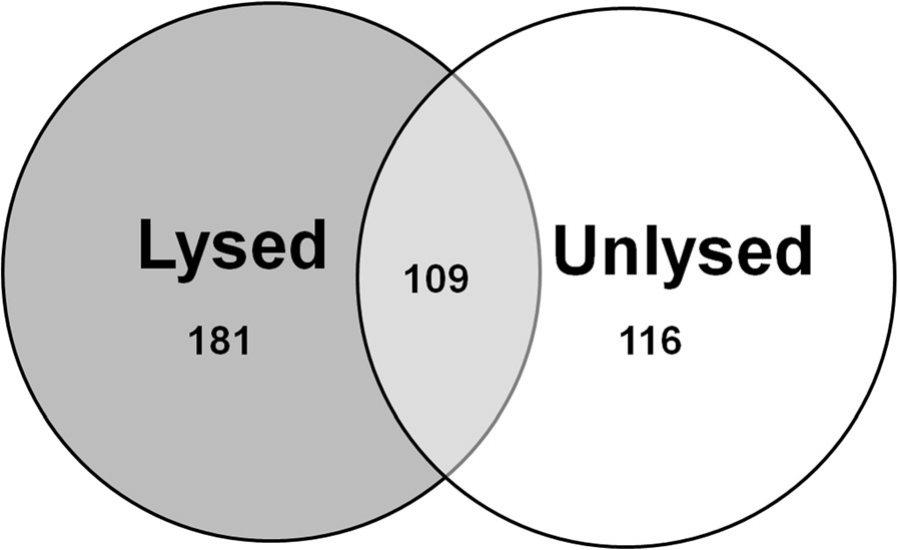
**Number of miRNAs detected in hemolysed and unlysed samples.** A Venn-diagram representing the total number of miRNAs detected in all (N = 10 per group) samples.

To determine the effects of hemolysis on serum miRNA profiles, we measured the serum miRNA profiles of lysed and unlysed serum samples from ten healthy volunteers. Hemoglobin levels of all samples were measured spectrophotometrically, all unlysed samples had a hemoglobin concentration below 0.1 g/l and all lysed samples had a hemoglobin concentration above 0.1 g/l (Table 1).Out of the 742 miRNAs profiled 109 miRNAs were detected in all 20 samples, 181 miRNAs were detected in all 10 hemolysed samples, and 116 miRNAs were detected in all 10 non-hemolysed samples. 36 miRNAs were detected in at least 5 of the 10 hemolysed samples at a Ct below 34 but were not detected in any of the matched non-hemolysed samples (Figure 2).

**Table 1:** Hemoglobin concentration

**Sample ID**	**Hemoglobin concentration (g/l) unlysed**	**Hemoglobin concentration (g/l) lysed**
11	0.051	0.552
12	0.048	1.217
14	0.055	0.620
15	0.056	0.220
16	0.042	0.154
17	0.074	1.091
18	0.069	1.048
19	0.075	2.428
20	0.037	1.235
21	0.070	2.249

Hemoglobin concentrations of matched lysed and unlysed serum samples.

To determine the best endogenous control to use for normalization we first identified 109 miRNAs that were present at detectable levels in all 20 samples. Because we were only interested in intra-individual comparisons, we then calculated the ratios of the raw Ct values of each miRNA in matched lysed and unlysed samples using the following formula: 2^( raw Ct (unlysed) − raw Ct (lysed)^. miR-122-5p and miR-1266-5p were the only miRNAs with ratios < 2 in all samples and had the lowest standard deviations of all miRNAs tested (Additional file 1: Table S1). Because miR-122-5p had the lowest average ratio and has been shown by others to be unaffected by hemolysis [20,21] it was used to normalize prior to fold-change analysis.

A synthetic spike-in (cel-miR-39-3p) was added and measured in 14 of the 20 samples to confirm that sample hemolysis did not significantly affect extraction efficiency. Table 2 shows the raw Ct value of the spiked-in RNA for all lysed and unlysed samples. There was no significant difference between the cel-miR-39-3p Ct values of lysed and unlysed samples.

**Table 2:** Synthetic spike-in levels in hemolysed samples

**Sample ID**	**cel-miR-39-3p raw Ct**	**cel-miR-39-3p raw Ct**
	**unlysed sample**	**lysed sample**
15	22.70	22.20
16	22.33	22.66
17	23.79	24.32
18	24.01	23.46
19	23.71	22.70
20	22.88	22.89
21	23.75	24.04

The raw Ct values of a synthetic spiked-in RNA (cel-miR-39-3p) for all lysed and unlysed samples demonstrates that hemolysis did not have a significant impact on RNA isolation efficiency.

A fold-change analysis identified 4 miRNAs that were up-regulated by at least 3-fold in all hemolysed samples and 231 miRNAs that were up-regulated by at least 3-fold in at least 1 hemolysed sample compared to unlysed samples. No miRNAs were down-regulated by at least 3-fold in more than 1 hemolysed sample. The spike-in was not significantly differentially expressed between hemolysed and non-hemolysed samples suggesting that the difference in miRNA levels is not due to the presence of inhibitors in the samples. Because clinical samples often contain some hemolysis [33], we have identified a list of miRNAs significantly affected by mechanical hemolysis to consider when conducting serum miRNA biomarker studies. For our purposes, we decided to exclude miRNAs up-regulated by at least 3-fold in at least 50% of samples or detected in 50% of hemolysed samples and not detected in matched unlysed samples leaving a final list of 162 miRNAs that are significantly influenced by hemolysis (Additional file 2: Table S2).

**Figure 3:**
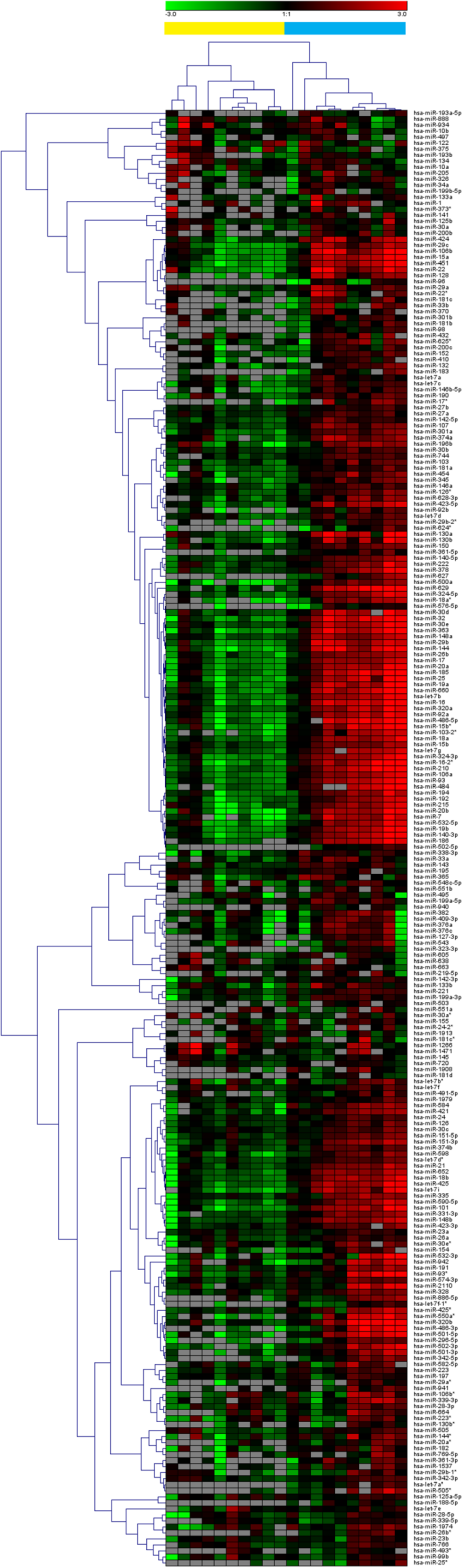
**The effect of hemolysis on serum miRNA levels.** An average-linkage Pearson correlation cluster analysis of the serum miRNA profiles of matched unlysed (yellow) and lysed (blue) samples collected from 10 healthy individuals. Only miRNAs detected in at least 80% of lysed samples or 80% of unlysed samples are included.

To measure the correlation between hemoglobin concentration and serum miRNA levels, the raw data was normalized to miR-99a-5p and miR-139-5p (rationale described below). Of the miRNAs detected in at least 80% of the hemolysed samples or 80% of the non-hemolysed samples, 177 miRNAs were significantly (corrected *P*-value < 0.05) correlated with hemoglobin concentration (Additional file 3: Table S3). A Pearson correlation cluster analysis of miRNAs show that the samples cluster according to hemoglobin concentration and not according to sample source (Figure 3).

### Selecting endogenous controls for serum miRNA studies

Currently, there are no standard endogenous controls for serum miRNA studies [32]. The varying levels of miRNAs observed in samples with high hemoglobin concentrations suggest that some current methods of normalization may be unreliable when samples exhibit hemolysis. Our data suggest that normalizing to liver specific miR-122-5p may be appropriate for matched samples as it is not affected by hemolysis and shows little variation in samples collected from the same person within a short period of time. However, because this miRNA is inconsistently expressed from person to person and has been shown to be deregulated under certain pathologies [34,35] it is not a suitable endogenous control for inter-individual comparisons. To identify a suitable endogenous control for serum miRNA studies, we examined the profiles of 154 serum samples from: 26 lung adenocarcinoma patients, 50 oral CIS/OSCC patients, 18 healthy non-smokers, 43 healthy smokers, and the 7 non-fasting and 10 hemolysed samples described in this study. Of the 46 miRNAs detected in all 154 serum samples, miRs 139-5p and 99a showed the lowest standard deviation across all samples even when the global mean values of these samples were included (Table 3). Therefore, for the following analyses, all data were normalized to the average Ct values of miR-99a-5p and miR-139-5p.

**Table 3:** Selecting an endogenous control

**miRNA**	Standard deviation*	**miRNA**	Standard deviation*
hsa-miR-139-5p	3.4E-10	hsa-miR-18b-5p	7.6E-09
hsa-miR-99a-5p	4.4E-10	hsa-miR-27b-3p	7.9E-09
hsa-miR-125a-5p	8.5E-10	hsa-miR-19a-3p	8.2E-09
hsa-miR-125b-5p	1.1E-09	hsa-miR-191-5p	8.2E-09
hsa-let-7d	1.2E-09	Global Mean	9.1E-09
hsa-miR-197-3p	1.2E-09	hsa-miR-150-5p	1.1E-08
hsa-miR-145-5p	1.5E-09	hsa-miR-107	1.2E-08
hsa-miR-140-5p	1.6E-09	hsa-miR-425-5p	1.4E-08
hsa-miR-342-3p	1.9E-09	hsa-miR-27a-3p	1.5E-08
hsa-miR-215-5p	1.9E-09	hsa-miR-142-3p	1.7E-08
hsa-miR-29a-3p	2.0E-09	hsa-miR-140-3p	2.1E-08
hsa-miR-378a-3p	2.1E-09	hsa-miR-122-5p	2.4E-08
hsa-miR-210-3p	2.2E-09	hsa-miR-23a-3p	2.5E-08
hsa-miR-146a-5p	2.2E-09	hsa-miR-103-3p	2.5E-08
hsa-miR-142-5p	2.2E-09	hsa-miR-185-5p	6.5E-08
hsa-miR-130a-3p	3.5E-09	hsa-miR-144-3p	9.5E-08
hsa-miR-1979	3.9E-09	hsa-miR-106a-5p	1.2E-07
hsa-miR-23b-3p	4.7E-09	hsa-miR-15a-5p	1.8E-07
hsa-miR-192-5p	4.9E-09	hsa-miR-20a-5p	2.0E-07
hsa-miR-30c-5p	5.0E-09	hsa-miR-92a-3p	3.0E-07
hsa-miR-29c-3p	5.0E-09	hsa-miR-223-3p	3.2E-07
hsa-miR-30b-5p	5.1E-09	hsa-miR-16-5p	9.7E-07
hsa-miR-222-3p	5.4E-09	hsa-miR-451a	4.9E-06
hsa-miR-424-5p	7.6E-09		

*All raw Ct values were converted to a linear scale (using 2^-Ct) before calculating standard deviation.

The standard deviation of 46 miRNAs detected in all of 154 serum samples. The global mean is included for comparison.

### The effects of fasting on serum miRNA profiles of healthy individuals

**Figure 4:**
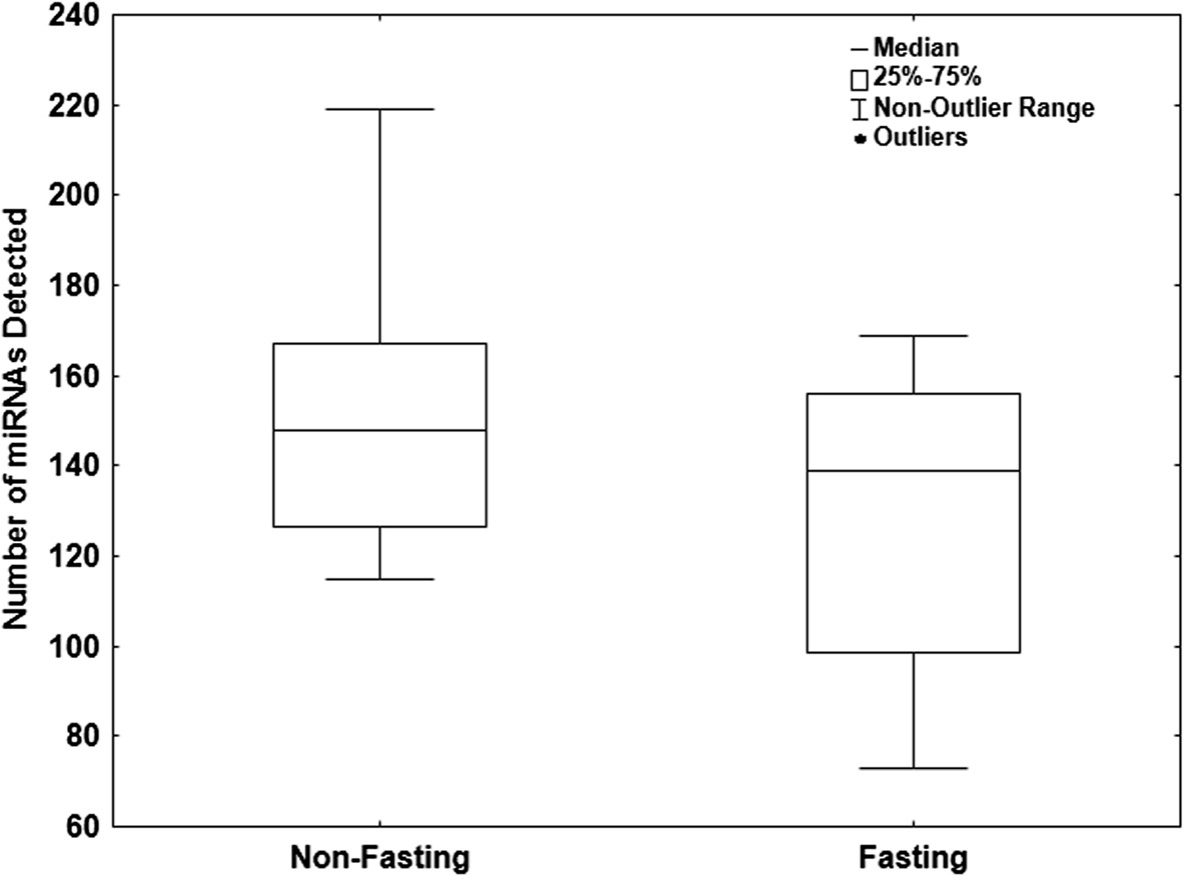
**Total number of miRNAs detected in fasting and non-fasting samples.** The total number of miRNAs (out of 742 profiled) present at detectable levels in fasting and non-fasting samples. *P*-value = 0.27, Mann-Whitney U test.

It has been suggested that fasting status may alter serum miRNA profiles due to lipids interfering with the RNA extraction [24] or to miRNAs from food entering circulation [25]. However, to our knowledge, this effect has not yet been tested. To assess the effects of fasting on serum miRNA profiles we collected blood samples from 7 healthy volunteers one hour after eating a fatty meal and again three weeks later, after fasting overnight. Serum triglyceride levels were measured enzymatically and were higher in the non-fasting samples (Table 4). A comparison of the total number of miRNAs detected in each sample showed that, after the exclusion of miRNAs significantly affected by hemolysis, the total miRNA count was slightly higher in the non-fasting samples compared to the fasting samples although not significant (P = 0.27, Figure 4). After normalization to miR-99a-5p and miR-139-5p, Pearson correlation coefficients between serum triglyceride concentration and miRNA levels showed 6 miRNAs were significantly (4 directly and 2 inversely) correlated with serum triglyceride levels (Table 5). However, a fold-change analysis between matched samples showed that no miRNAs were significantly (by at least 3-fold in at least 50% of cases) differentially expressed between the fasting and non-fasting samples. No miRNAs were detected in over 3 of the 7 fasting or non-fasting samples at a Ct below 34 and not detected in the corresponding matched sample. A Mann-Whitney U test of miRNAs normalized to miR-99a-5p and miR-139-5p expressed in at least 5 of 7 fasting or 5 of 7 non-fasting samples showed that no miRNAs were significantly (P < 0.05) differentially expressed between the two groups (data not shown).

**Table 4:** Triglyceride concentration in serum

**Sample**	**Triglyceride concentration (mg/dl) non-fasting**	**Triglyceride concentration (mg/dl) fasting**
1	202.8	120.6
2	152.1	96.2
3	148.5	52.1
4	93.3	30.4
5	176.4	102.4
6	72.4	57.8
7	53.5	45.7

Triglyceride concentration of fasting and non-fasting serum samples.

**Table 5:** Serum miRNA correlation with triglyceride levels

**miRNA**	**Correlation coefficient (R)**	**P-value**
hsa-miR-17-3p	0.88	2.9E-05
hsa-miR-375	0.70	0.005
hsa-miR-328-3p	−0.64	0.013
hsa-miR-223-3p	−0.57	0.033
hsa-miR-593-3p	0.57	0.034
hsa-miR-130b-5p	0.55	0.040

6 miRNAs significantly (P <0.05) correlated with serum triglyceride levels and corresponding Pearson correlation coefficients.

### The effects of smoking on serum miRNA profiles

**Figure 5:**
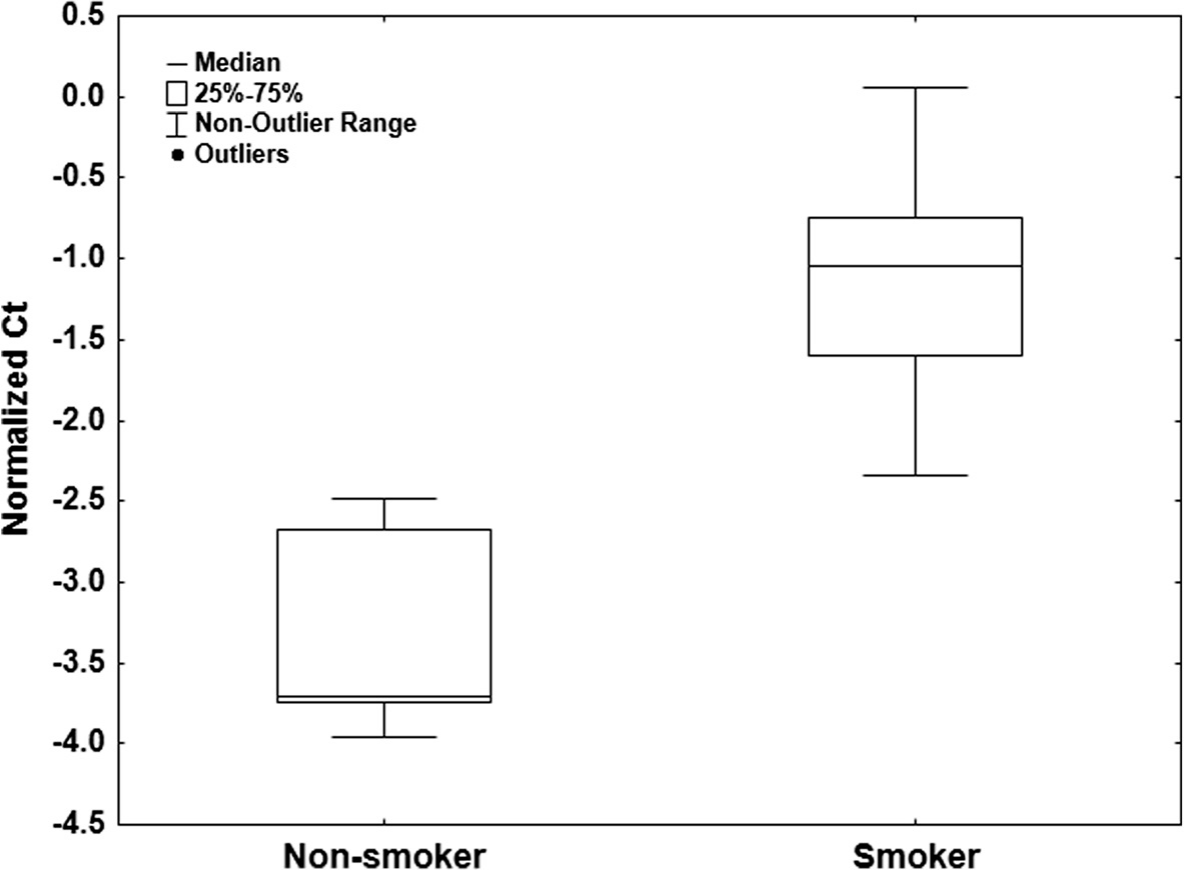
**Serum miR-128-3p expression in current smokers and never smokers.** Levels of miR-128-3p in the serum of non-smokers and current smokers. Uncorrected *P*-value = 0.004, Corrected *P*-value = 0.78, Mann-Whitney U test.

To examine the effects of tobacco smoke on serum miRNA expression we compared the profiles of 10 current smokers to 10 age and sex-matched controls (Table 6). A Mann-Whitney U test conducted on miRNAs normalized to miR-99a-5p and miR-139-5p and detected in at least 80% of smokers or 80% of non-smokers showed that no miRNAs were significantly (corrected *P*-value < 0.05) differentially expressed. However, miR-128-3p showed the lowest uncorrected *P*-value (0.001) and this miRNA was detected in 7 non-smoker cases and 9 smoker cases and was slightly up-regulated in smokers (Figure 5). No miRNAs were present in only smoking samples or only non-smoking samples.

**Table 6:** Patient demographics

	**Current-smokers**	**Non-smokers**
Males	3	3
Females	7	7
Median age	60.5	52
Age range	52-67	40-64
Median pack year	46.3	0

Patient demographics for smoking/non-smoking samples.

### Changes in serum miRNA levels over time

To assess changes in serum miRNA levels in healthy individuals over time we compared the profiles of two samples collected from the same individual over varying time periods (Table 7). Pearson correlation coefficients of the levels of miRNAs detected in at least 19 of 24 samples showed a strong correlation between samples collected over different time periods from the same person (Table 7). A fold-change analysis showed no miRNAs were frequently significantly deregulated between matched samples (up or down-regulated by at least 3-fold in at least 50% of cases). Nine miRNAs (miR-888-5p, miR-454-3p, miR-10a-5p, miR-181c-5p, miR-1909-3p, miR-20a-3p, miR-484, miR-501-5p, and miR-622) were detected in at least 50% of cases and not detected in the corresponding matched control. Of these nine miRNAs, four (miR-501-5p, miR-454-3p, miR-20a-3p, and miR-484) are also in the list of 162 miRNAs significantly affected by hemolysis and therefore the difference in miRNA levels could be due to differences in the amount of hemolysis in each sample.

**Table 7:** Correlation between samples collected at different time points

**Sample**	**Correlation**^**1 **^**coefficient**	**Time between sample collection**
1	0.88	13 months 7 days
2	0.99	15 Months 7 days
3	0.99	17 months 3 days
4	0.99	11 months 9 days
5	0.98	7 months 10 days
6	0.99	5 months 3 days
7	0.99	13 months
8	0.99	12 months 19 days
9	0.95	2 months 14 days
10	0.99	6 months 7 days
11	0.99	10 months
12	0.87	11 months 22 days

1. Calculated using miRNAs expressed in at least 19 of 24 samples

Pearson correlation coefficients for miRNA profiles of serum collected at two different time points from healthy individuals.

## Discussion

An ideal biomarker can be measured non-invasively and is highly sensitive and specific to the disease state of interest. The stability of circulating miRNAs and their differential expression in the serum and plasma of patients with cancer and other conditions make these molecules attractive biomarkers. However, in order for a biomarker to have a high positive predictive value it should not be significantly influenced by technical variables or pathologic conditions unrelated to the disease being investigated. In this study we examined the effects of a range of technical and individual factors on serum miRNA levels. Our results have identified a list of miRNAs that are significantly affected by these factors and are therefore likely to have very limited utility as biomarkers of disease.

We have contributed to previous studies examining the role of blood cell contaminants in serum miRNA quantification [19-21]. While Kirschner et al have performed a similar study in plasma [23] and miRNA profiles of blood cells and blood microvesicles have been previously identified [19], to our knowledge, this is the first study specifically examining the effect of mechanical hemolysis on the quantification of 742 miRNAs in matched serum samples. It should be noted that we did not distinguish between microRNAs in total serum and microRNAs in circulating microvesicles (such as exosomes). that Other studies examining the effects of hemolysis on circulating miRNA profiles have done so by profiling blood cells directly or by adding hemolysate back into already separated serum or plasma samples [20]. However, Dimeski (2004) demonstrated that samples prepared in this way can have a different effect on analytes than samples prepared by mimicking mechanical hemolysis [29]. By mimicking hemolysis caused by improper blood collection or preparation we have identified a list of 162 miRNAs that are most likely to be affected by hemolysis in a clinical setting. Currently, researchers examining the role of hemolysis on plasma and serum miRNA profiles have suggested excluding hemolysed samples from studies [22]. This method may be useful in identifying biomarkers because it allows the measurement of all miRNAs while avoiding the effects of hemolysis. However, from a clinical standpoint, excluding samples with even low (~0.15 g/l) levels of hemolysis is impractical because the frequency of hemolysis in a clinical setting has been reported as being as high as 5.6% and samples with low amounts of hemolysis cannot be identified visually [36]. Asking these patients to return for multiple blood draws requires further staff time and supplies and can delay diagnosis leading to increased stress on the patient [37]. Therefore, we propose that rather than excluding hemolysed samples from biomarker studies, researchers should eliminate miRNAs significantly affected by hemolysis when identifying potential biomarkers.

Several groups have addressed the importance of assessing analytical parameters for serum miRNA studies especially with regards to comparing results from multiple groups [20,38]. In this study we have identified potential endogenous controls that show consistent expression across 154 samples including samples from cancer patients as well as samples exhibiting hemolysis. In cases with samples that may be compromised by hemolysis, this method is more appropriate than some commonly used normalization methods. For example, normalizing to the geometric mean of miRNAs expressed in all samples [39] can produce skewed results if a sample is contaminated with miRNAs from blood cells. This occurs because more highly expressed serum miRNAs, which are used for global mean normalization, are also expressed in blood cells and will be present at higher levels in hemolysed samples [21]. Therefore, samples exhibiting hemolysis will appear to have high RNA input due to an overall lower normalization value and miRNAs that are not expressed in blood cells will seem to be down-regulated in that sample. Another commonly used method for normalization is the use of miR-16-5p as an endogenous control [40]. However, we have demonstrated that the levels of serum miR-16-5p are significantly increased in samples with high hemoglobin concentrations suggesting that this method is also inappropriate in cases where some samples may be contaminated with blood cell miRNAs. Finally, another commonly used method for miRNA normalization is the use of U6 as an endogenous control. U6 is a nuclear RNA and has been used to normalize tissue and cell miRNA expression [41]. However, because U6 is localized in the nucleus it should not be present at consistent, high levels in the non-cellular components of blood [32]. Indeed, of the 154 samples analyzed in this study U6 is only present at a detectable level in 84 samples suggesting U6 is not a suitable endogenous control for serum miRNA studies. We propose that miR-99a-5p and miR-139-5p should be used for sample normalization instead of these commonly used endogenous controls due to the fact that they are not significantly affected by hemolysis and that they have the lowest standard deviation across a series of 154 samples (Table 3).

A recent report has shown that plant miRNAs are present in the sera of humans and that these miRNAs are acquired through food intake [25]. This finding suggests that miRNAs acquired through food intake would show variable expression over time and could be affected by fasting status. Furthermore, fasting status alters the amount of lipids in the blood which could interfere with RNA extraction leading to variable miRNA levels [24]. Here we show that there is no significant difference in the total number of miRNAs detected in fasting versus non-fasting samples demonstrating that the presence of lipids in the blood does not lead to a loss of less abundant miRNAs during extraction. Furthermore, no miRNAs examined in our assay were significantly differentially expressed between fasting and non-fasting samples indicating that fasting status will not interfere with serum miRNA biomarker discovery in subjects with normal miRNA physiology. However, 6 miRNAs did show a significant correlation with serum triglyceride levels suggesting some miRNAs may be slightly (but not significantly) influenced by serum lipids. There are limitations to our study including a small sample size and the fact that we only examined short term dietary effects on circulating miRNAs. Therefore, experiments with long-term, controlled diets and large sample sizes need to be carried out in order to rigorously test the overall effects of diet on serum miRNA profiles [42].

As smoking plays an important role in the development of lung and other cancers, it must be considered when identifying potential biomarkers for these diseases. Therefore, we have also examined the influence of smoking on serum miRNA profiles. Because no miRNAs were significantly differentially expressed between smokers and non-smokers our results suggest that smoking status will not interfere with serum miRNA biomarker studies. A recent study using 6-month cigarette smoking in mice found that the levels of miR-128-3p were significantly changed in the lung tissue and plasma of exposed mice suggesting a role for this miRNA in the cellular response to cigarette smoke exposure [43]. We have found that although miR-128-3p was not significantly deregulated in the serum of smokers, this miRNA was detected in more current smoker samples than non-smoker samples and had the lowest P-value prior to correction. Taking the results of both studies into account, the role of smoke exposure should be considered when determining the possible utility of miR-128-3p as a biomarker of disease.

When identifying biomarkers for disease detection and/or monitoring, an important aspect to consider is the possibility of changes in biomarker levels over time in the healthy population. In order to evaluate serum miRNA variability in healthy individuals, we measured the levels of miRNAs in two serum samples taken from 12 individuals over varying time periods. Pearson correlation coefficients of the matched samples show no pattern between correlation coefficient and time between sample collections. This lack of a pattern and the fact that the matched samples with the lowest R-value (0.87) are still highly correlated suggest that, up to 17 months, overall serum miRNA levels show little variability in healthy individuals. Further, long-term, studies are required to determine changes in serum miRNAs over several years. A fold-change analysis showed no individual miRNA was consistently deregulated in these matched samples implying that variability in serum miRNAs over time should not interfere with biomarker studies examining abundant serum miRNAs. Among the less abundant miRNAs, 9 were differentially detected in the matched samples. This result demonstrates that these less abundant miRNAs are not suitable biomarkers due to their inconsistent expression.

## Conclusions

Although this study has limitations such as small sample sizes, the results presented here identify factors that should be taken into consideration when selecting endogenous controls and biomarker candidates. In order for serum miRNA biomarkers to eventually be implemented in a clinical setting, pre-analytical and analytical variables affecting serum miRNA profiles should be examined and standardized. In addition to the variables described in this study other areas requiring further examination include the effect of inflammation, hormonal activity, and diurnal variation on serum miRNA profiles of healthy individuals as well as analytical factors such as the best platform and statistical tests to use for the detection and determination of differentially expressed miRNAs. Once these variables are well defined, the complete potential of circulating miRNAs as biomarkers can be fully explored.

## Competing interests

The authors declare they have no competing interests.

## Authors’ contributions

SAM carried out all experiments and drafted the manuscript. CM and SAM conducted data analyses. SL was responsible for clinical interpretations and sample collection. CG was responsible for overall supervision. All authors contributed equally in study design and have approved the final manuscript.

## Pre-publication history

The pre-publication history for this paper can be accessed here:

http://www.biomedcentral.com/1472-6890/14/27/prepub

## Supplementary Material

Additional file 1: Table S1 Raw Ct ratios. The ratios of the raw Ct values of each miRNA in matched lysed and unlysed samples using the following formula: 2^- raw Ct (lysed)/2^- raw Ct (unlysed). Click here for file

Additional file 2: Table S2 miRNAs significantly affected by hemolysis. A list of miRNAs A) significantly up-regulated (by ≥3-fold) in hemolysed serum samples or B) detected in hemolysed samples at a Ct ≤34 but not detected in the corresponding matched control. Click here for file

Additional file 3: Table S3 miRNAs significantly correlated with serum hemoglobin. 177 miRNAs significantly correlated with hemoglobin concentration and corresponding Pearson correlation coefficients. Click here for file
